# Supplementary material for: Isoform-level transcriptome-wide association uncovers genetic risk mechanisms for neuropsychiatric disorders in the human brain
Source: Nat Genet. 2023 Nov 30;55(12):2117–28. doi: 10.1038/s41588-023-01560-2 (PMC10703692; doi:10.1038/s41588-023-01560-2)
Supplement: Supplementary file 1 — Supplementary Note, Methods, Figures 1–4, Table and Data Legends and References. [file 41588_2023_1560_MOESM1_ESM.pdf]

nature genetics

Article

https://doi.org/10.1038/s41588-023-01560-2

Isoform-level transcriptome-wide association uncovers genetic risk mechanisms for neuropsychiatric disorders in the human brain

In the format provided by the authors and unedited

Isoform-level transcriptome-wide association uncovers novel genetic risk mechanisms for neuropsychiatric disorders in the human brain

Supplementary Information

Supplementary Note

Here, we discuss added evaluations of isoTWAS and TWAS: (1) across different factors that may influence prediction and (2) when dominant isoforms are failed to be detected. We also include a brief discussion of some example genes prioritized by isoTWAS in our analysis of 15 neuropsychiatric traits.

Gene and isoform expression prediction across different factors

We evaluated the prediction of multivariate isoform-centric prediction models across a variety of factors that may influence the genetic architecture of isoform regulation at a locus or the inference of the regulation. In general, we computed three ratios: (1) the isoform prediction ratio, or the ratio of number of isoforms that are predicted at CV $R^2 > 0.01$ using multivariate and univariate models, (2) the inclusion criterion ratio, or the ratio of the number of genes that meet inclusion criteria for isoTWAS (gene with 1+ isoforms predicted at CV $R^2 > 0.01$) compared to TWAS (gene is predicted at CV $R^2 > 0.01$), and (3) the gene prediction ratio, or the ratio of the number of genes that are predicted at CV $R^2 > 0.01$ using isoform-centric isoTWAS models compared to gene-centric TWAS models. We plot boxplots of these ratios across the 48 GTEx tissues, and vary these factors across bins. In general, we note that, despite trends in these ratios across these factors, the ratios are always above 1, reinforcing the gains the prediction afforded by the multivariate isoform-centric prediction in isoTWAS.

1. *Number of expressed isoforms per gene (***Extended Data Fig. 6a***).* The isoform prediction ratio stays relatively even as the number of isoforms per gene increases. The inclusion criterion ratio increases as the number of isoforms per gene increases but only until approximately 10 isoforms per gene. For genes with >10 isoforms per gene, the inclusion criterion ratio remains relatively even. There is a clear increasing trend in the median number of well-predicted isoforms per gene (CV $R^2 > 0.01$) as the number of expression isoforms per gene increases, suggesting that the increased number of isoforms per gene provides more information about shared genetic architecture between isoforms that can be leveraged for improved prediction. Lastly, we see a similar increasing trend in the gene prediction ratio as the number of isoforms per gene increases (increase in the ratio until approximately 10 isoforms per gene and a leveling off after).

2. *Maximum isoform fraction per gene* (**Extended Data Fig. 6b**). We computed, using the raw counts of isoforms, the isoform fraction of each isoform of a gene using the isoformtoIsoformFraction() function in the Bioconductor package IsoformSwitchAnalyzeR[1]. This function computes the fraction of each isoform's expression to the total gene expression. We then found the isoform with the maximum isoform fraction for each gene and termed this fraction the maximum isoform fraction for the gene. In general, genes with large maximum isoform fraction are dominated by a single isoform, whereas genes with a small maximum isoform fraction have multiple isoforms with similar levels of expression.

We find that, as maximum isoform fraction increases, there is a slight increase in the isoform prediction ratio, a larger increase in the inclusion criterion ratio, and no general trend in the gene prediction ratio.

3. *Gene length* (**Extended Data Fig. 6c**). We computed the length of each gene as the difference in the end and start positions of the gene, as annotated in Ensembl v109. We find that, as gene length increases, there is a slight increase in both the isoform prediction ratio and the inclusion criterion ratio, and no general trend in the gene prediction ratio.

4. *SNP density* (**Extended Data Fig. 6d**). We computed the number of SNPs that are within 1 Mb of the gene body, calling this value the SNP density of the gene locus. The SNP density represents the number of SNPs that comprise the design matrix in both the isoform-centric isoTWAS and gene-centric TWAS prediction models. We find that shows that, as SNP density increases, there is a slight decrease in the isoform prediction ratio but no general trend in the inclusion criterion ratio and gene prediction ratio.

5. *Sample size* (**Extended Data Fig. 6e**). We find that, as sample size increases, there is a decrease in the gene prediction ratio. This may reflect that, with larger sample sizes, gene-level expression QTLs may start to reflect the more subtle isoform-level expression QTLs, leading to a decrease in this ratio. We do note that, even at the largest sample sizes in GTEx, this gene prediction ratio is greater than 1. In datasets of small sample size (<175 samples), the isoform prediction ration and inclusion criterion ratio are largest. These ratios decrease in datasets of larger sample size, but the ratio does not decrease consistently (e.g., isoform prediction ratio is higher in datasets of 250-500 samples compared to 175-200 and inclusion criterion ratio remain relatively similar as sample size increases beyond 175).

6. *Proportion of shared isoTWAS model effect SNPs* (**Extended Data Fig. 6f**). For each isoform's predictive model, we determined which SNPs have large effects. First, we standardized the effect sizes in the model to mean 0 and unit variance. We found the SNPs whose effect sizes deviated significantly (Benjamini-Hochberg adjusted $P < 0.05$) and called them the isoform's effect SNPs. For each gene, we then computed the proportion of effect SNPs that were shared across all isoforms of the same gene. We also find a clear increasing trend in the isoform prediction ratios, indicating that multivariate modelling can leverage shared isoform QTL architecture to improve marginal prediction of each isoform's expression. The inclusion criterion ratio remains relatively even as this proportion increases. The expression prediction ratio shows a decreasing trend as the proportion of shared isoTWAS effect SNPs increases, reflecting results from simulation (**Fig. 2**).

7. *Mean normalized counts* (**Extended Data Fig. 6g**). Here, using the isoform and gene counts normalized to library size and gene length, we compute each isoform and gene's mean normalized count across samples, using the countsFromAbundance = 'lengthScaledTPM' option from tximport[2]. Since the bins of mean normalized counts for gene and isoform expression do not map one-to-one as for the previous factors, we only compute and plot the isoform prediction and gene prediction ratios. As the mean normalized counts for isoform expression increases, we see a decreasing trend in the isoform prediction ratio, with a slight increase in the bin of isoforms with large mean normalized counts. We see no clear trend in the gene expression prediction as the mean normalized gene counts increase, though we see a similar increase in the largest bin.

8. *Quantification variance across inferential replicates of genes and isoforms* (**Extended Data Fig. 6h**). Here, using the raw isoform and gene counts, we compute the quantification variance across inferential replicates from Salmon[3]. We obtain the 50 inferential replicated from Salmon and import this using the Bioconductor package tximport[2]. We then computed the quantification variance for each isoform and gene using the computeInfRV() function from the Bioconductor package fishpond[4]. Briefly, this function first computes a matrix of variance (samples by features) across the 50 inferential replicates. Then, it computes the inferential quantification variance as the difference of the variance matrix and the mean counts matrix (Salmon Expectation-Maximization point estimates), standardized by the mean counts matrix. We collapse this inferential quantification variance to a per-feature measure by taking the mean of each row in the inferential quantification variance matrix. Again, since the bins

of quantification variance for gene and isoform expression do not map one-to-one as for the previous factors, we only compute and plot the isoform prediction and gene prediction ratios. We find, for isoforms with low quantification variance (variance < 1.5), the isoform prediction ratio stays relatively even but increases as isoform quantification variance exceeds 1.5. However, there is no general trend with gene prediction ratio as gene quantification variance increases. Leveraging this quantification variance to improve prediction is an interesting and worthwhile methodological opportunity that is discussed in the Discussion section.

Synthetic leave-one-isoform-out models

We consider a corollary experiment to assess how well isoTWAS prediction models impute gene and isoform expression when isoforms are failed to be detected. Across the 13 brain tissues in GTEx, we selected a random set of ~9000 genes in the following manner:

- We stratified our gene sets by the number of isoforms per gene and subset to genes with between 3 to 16 isoforms.
- We then randomly selected 50 genes from each group of genes with a certain number of isoforms per gene.

Then, we generated a synthetic leave-one-isoform-out (LOO) dataset, where the dominant isoform of each gene is missing. Using the raw transcript-level salmon quantifications, we removed each gene's dominant isoform and summarized gene expression using tximeta's summarizeToGene() function[4]. Then, we trained isoTWAS and TWAS models in the synthetic datasets and imputed isoform (using isoTWAS) and gene (using isoTWAS and TWAS) expression in the original GTEx datasets. We compared these leave-one-out predictions to predictions from models trained in the original GTEx datasets.

Extended Data Fig. 3d plots the distribution of the percent difference in isoform expression (using multivariate compared to univariate models), when using the original and synthetic leave-one-out training datasets. We find that the advantage of the multivariate models over univariate models greatly decreases with using this leave-one-out dataset compared to the true original, true gene expression measures. In addition, this drop in performance is consistent as we increase the number of isoforms per gene in the original dataset. This result is unsurprising as, in the leave-one-out dataset, though each isoform's expression remains equal to its expression in the original dataset, the correlation structure is altered. The multivariate models depend on shared genetic architecture to best predict each isoform's expression marginally. **Extended Data Fig. 3d** plots the distribution of the percent difference in gene expression (using isoTWAS compared to TWAS models), when using the original and synthetic leave-one-out training datasets. We find that the advantage of the isoTWAS models over TWAS models greatly decreases with using this leave-one-out dataset compared to the true original, but only for genes with smaller numbers of isoforms per gene. As the number of isoforms per gene in the original dataset increases, this drop in performance decreases. In general, these results suggest that, when a large portion of the total gene's expression is removed from the dataset, both isoform- and gene-centric expression prediction is negatively affected. Taken together, these results about isoforms underscores that proper quantification of isoforms is important, especially when building a genetic predictor, and motivates a need to develop more accurate and updated transcriptome annotations that are tissue-specific to aid isoform expression quantification. We discuss this further in the Discussion section.

High pLI genes identified by isoTWAS but not TWAS

We discuss some examples of high pLI genes that were identified by isoTWAS. For example, ENST00000519133 (*SNAP91* isoform in 6q14.2) associated with SCZ in developmental cortex (adjusted P = 6.06×10^{-7} in isoTWAS, pLI = 0.99), with a GWAS-significant variant rs217291 within the gene

body. *SNAP91* affects clathrin and phosphatidylinositol biding activity and synaptic vesicle recycling and impacts synaptic development[5]. In addition, imputed developmental cortex expression of ENST00000476671 (*KMT2E* isoform in 7q22.3) was associated with CDG risk (adjusted P = 7.63x10^{-3}, pLI = 1); the GWAS SNP rs2385537, associated in a meta-analysis of ADHD, ASD, BP, and SCZ[6], is within the gene body. *KMT2E* regulates post-translational histone methylation of histone 3 on lysine 4A, and *KMT2E* heterozygous variants are associated with risk of neurodevelopmental disorders[7]. Imputed adult brain expression of ENST00000492146 (*SFMBT1* isoform in 3p21.1, adjusted P = 1.14x10^{-6}, pLI = 0.94) associated with SCZ risk and contains a GWAS SNP within the gene body (rs2071044). In a recent gene-based analysis of GWAS data for SCZ and BD, decreased *SFMBT1* expression associated with increased risk of both disorders[8], consistent with the effects for the isoform we identified. Lastly, ENST00000537270 (*KMT5A* isoform in 12q24.31, adjusted P = 1.06x10^{-11}, pLI = 0.99) associated with SCZ risk. *KMT5A*, a H4K20 methyltransferase, has been previously implicated in GWAS for SCZ but cis-eQTLs of the gene have not be colocalized with the GWAS signal[9–11].

Supplementary Figures

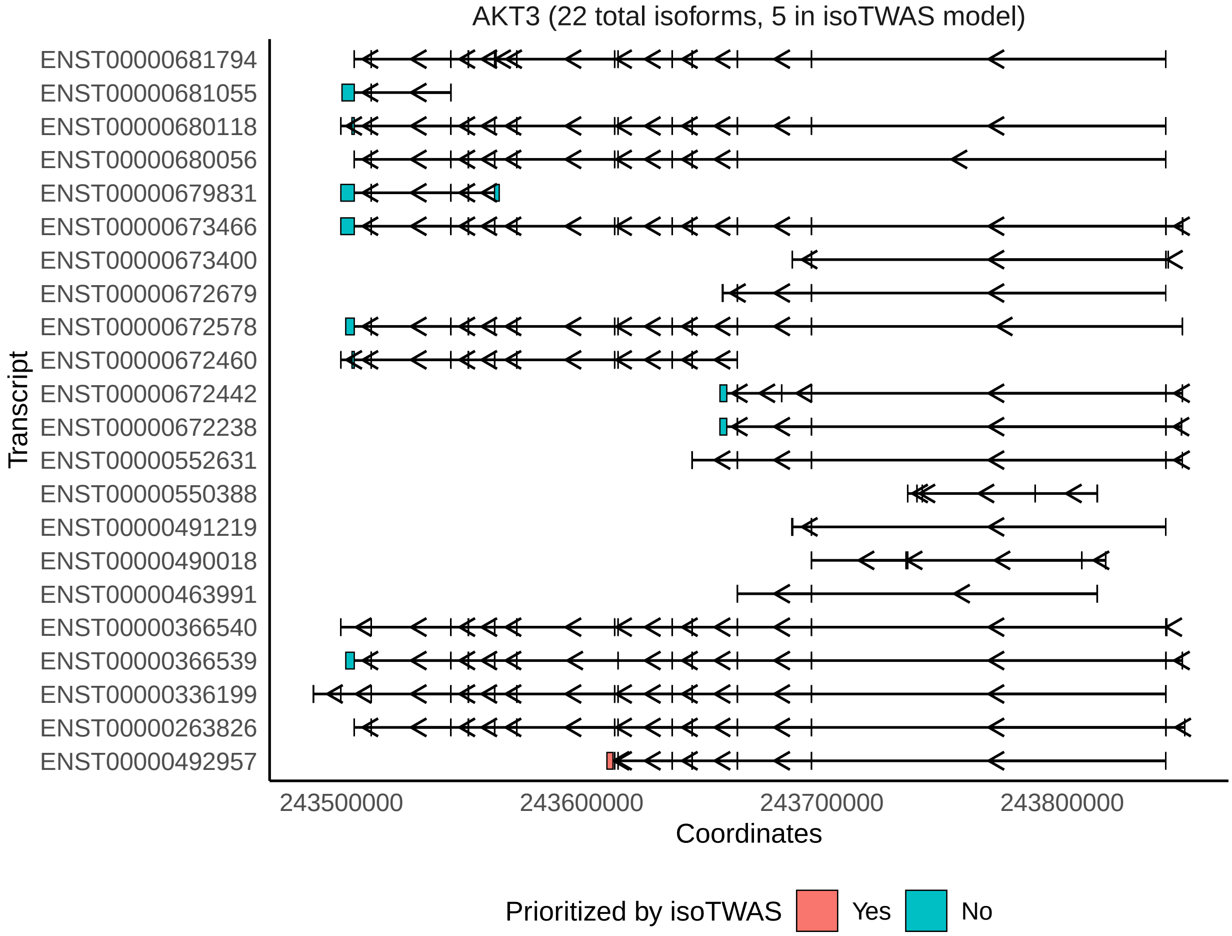

Figure S1: Comparison of exon and intron structure of *AKT3* isoforms, based on Gencode v38 reference. The effect isoform ENST00000492957 in **Fig. 6** is indicated in pink and is at the bottom.

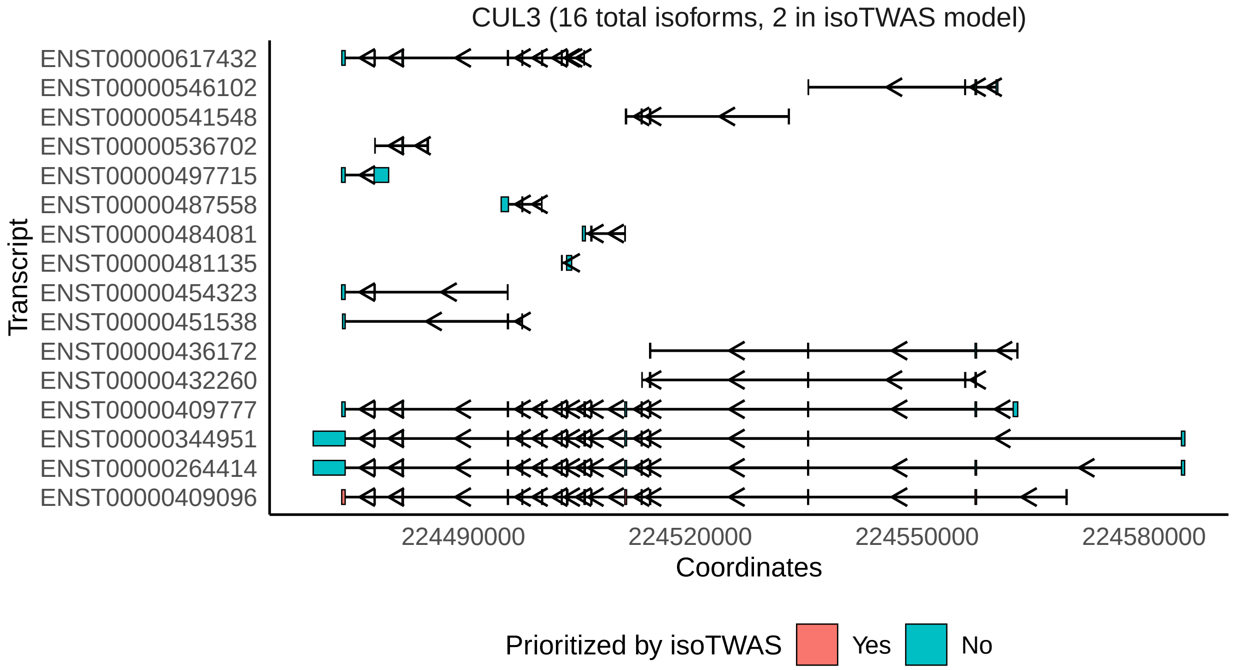

Figure S2: Comparison of exon and intron structure of *CUL3* isoforms, based on Gencode v38 reference. The effect isoform ENST00000409096 in **Fig. 6** is indicated in pink and is at the bottom.

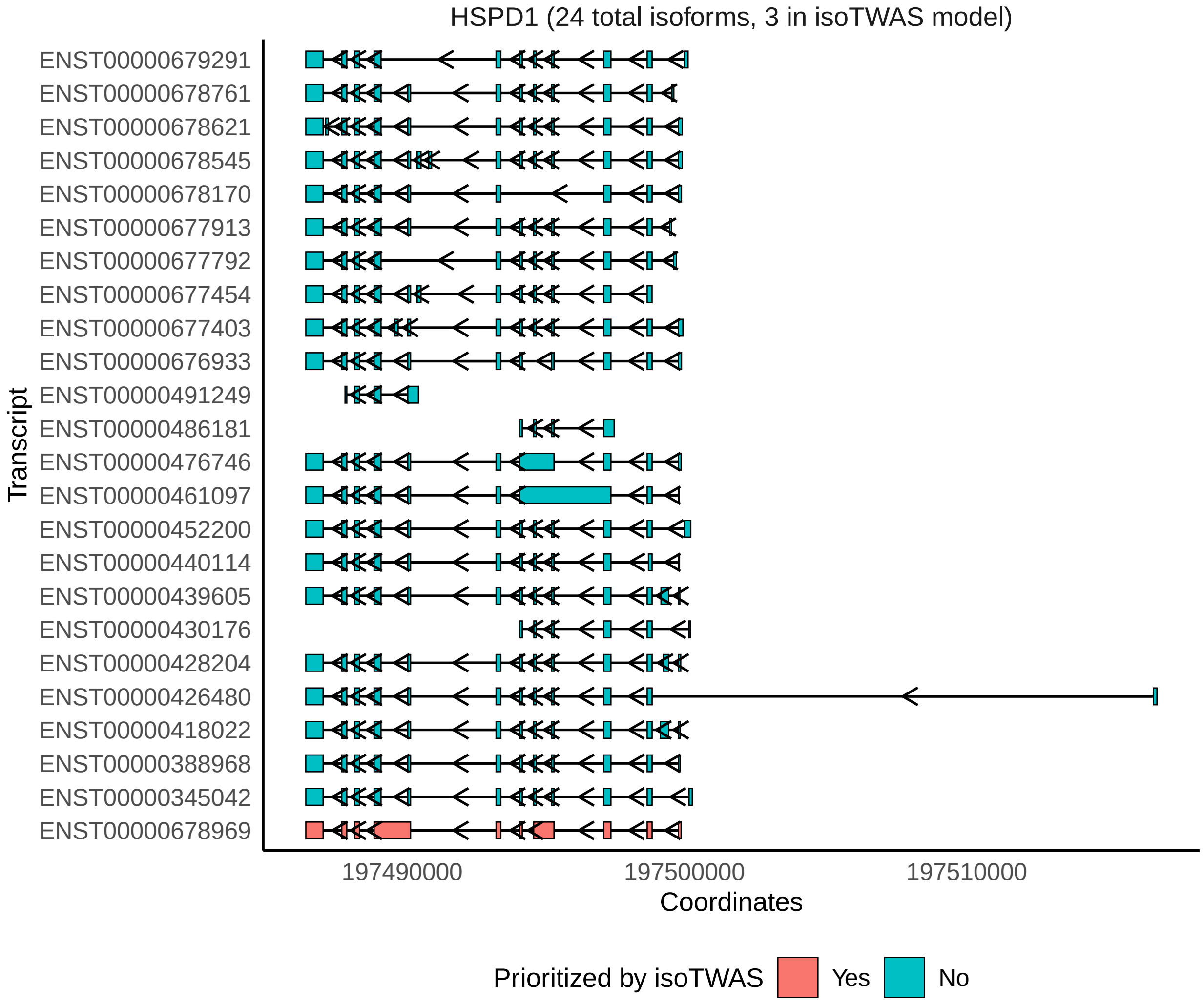

Figure S3: Comparison of exon and intron structure of *HSPD1* isoforms, based on Gencode v38 reference. The effect isoform ENST00000678969 in **Fig. 6** is indicated in pink and is at the bottom.

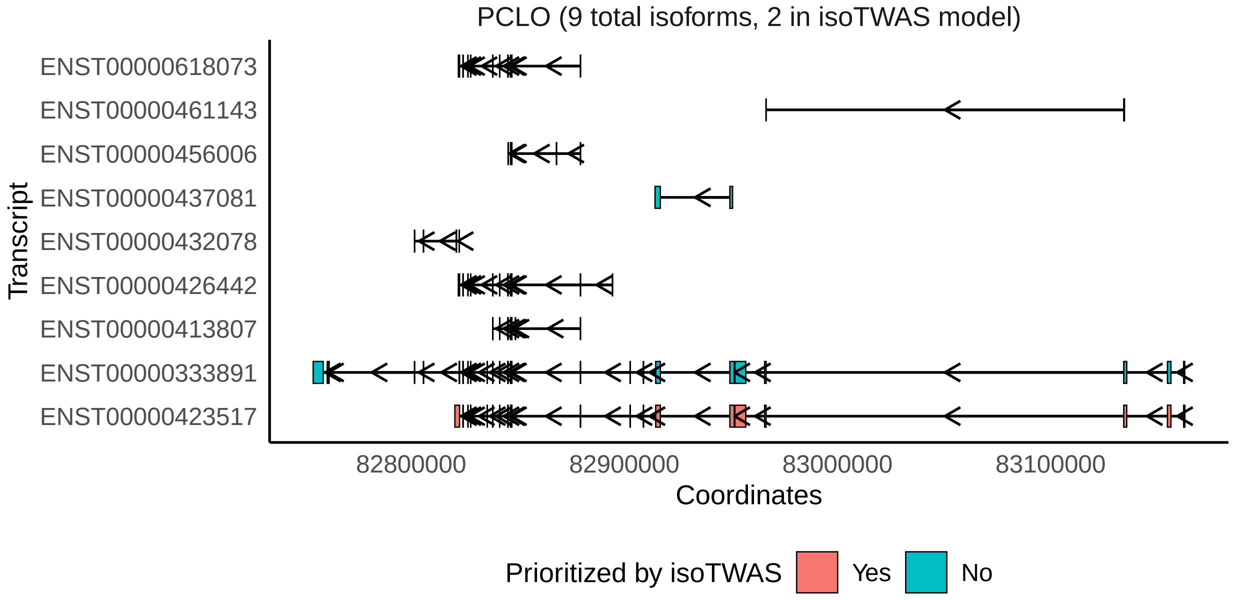

Figure S4: Comparison of exon and intron structure of *PCLO* isoforms, based on Gencode v38 reference. The effect isoform ENST00000423517 in **Fig. 6** is indicated in pink and is at the bottom.

Supplementary Methods

Here, we describe mathematical details of the predictive and hypothesis testing methods in the isoTWAS pipeline.

Predictive modeling

For a gene G with M isoforms across N samples, with expression measured across R inferential replicates, we consider the following multivariate linear model:

$$\mathbf{Y}_G^* = \mathbf{X}_G \mathbf{B}_G + \mathbf{E}_G, \tag{1}$$

where

- $\mathbf{Y}_G^*$ is the $N \times M$ matrix of isoform expression for gene G,
- $\mathbf{X}_G$ is the $N \times P$ matrix of genotype dosages (coded as 0,1, or 2 alternative alleles at a SNP) for SNPs within a *cis*-window of the body G,
- $\mathbf{B}_G$ is the $P \times M$ matrix of SNP effects on isoform expression, and
- $\mathbf{E}_G$ is a matrix of random errors, such that $\text{vec}(\mathbf{E}_G) \sim N_{NM}\left(0, \mathbf{\Sigma} = \mathbf{\Omega}^{-1} \otimes \mathbf{I}_N\right)$. Here, $\mathbf{\Sigma}$ is the variance-covariance matrix of the random errors, with $\mathbf{\Omega} = \mathbf{\Sigma}$ representing the precision matrix. The columns of $\mathbf{X}_G$ can be standardized to mean 0 and variance 1 to remove the intercept term from the model.

We implement 4 different multivariate methods to estimate $\hat{B}_G$.

Multivariate elastic net

Multivariate elastic net is an extension of elastic net regression for a multivariate response variable. The optimization here, fit through coordinate descent, solves

$$\text{argmin}_{\mathbf{B}_G} \left\{ \frac{1}{2N} \sum_{i=1}^{N} \|y_i - \mathbf{B}_G^T x_{G,i}\|_F^2 + \lambda \left[(1-\alpha)\|\mathbf{B}_G\|_F^2/2 + \alpha \sum_{j=1}^{P} \|\beta_{G,j}\|_2 \right] \right\}.$$

Here, $\beta_{G,j}$ is the jth row of the SNP effects matrix $\mathbf{B}_G$. There is a group-lasso penalty on each M-length vector of isoform effects for a single SNP. This penalty works on the whole group of coefficients for each response: either all coefficients are 0, or none are 0. All coefficients are shrunk by the λ penalty, optimally selected through cross-validation. Intuitively, multivariate elastic net should be optimal in settings where the causal isoQTLs are the shared across different isoforms of the same gene. We fit this model using the `glmnet` package in `R`[12] for the mixing parameter $\alpha \in \{0, .5, 1\}$.

Multivariate Regression with LASSO with Covariance Estimation

From Equation 1, we jointly estimate $\mathbf{B}_G$ and $\mathbf{\Omega}$ by minimizing the following objective function:

$$\left(\hat{\mathbf{B}}_G, \hat{\mathbf{\Omega}}\right) = \text{argmin}_{\mathbf{B}_G, \mathbf{\Omega}} \left\{ g(\mathbf{B}_G, \mathbf{\Omega}) + \lambda_1 \sum_{j' \neq j} |\omega_{j',j}| + \lambda_2 \sum_{j=1}^{p} \sum_{k=1}^{q} |b_{jk}| \right\},$$

where

$$g(\mathbf{B}_G, \mathbf{\Omega}) = \text{tr}\left[n^{-}1(\mathbf{Y}_G^* - \mathbf{X}_G\mathbf{B}_G)^T(\mathbf{Y}_G^* - \mathbf{X}_G\mathbf{B}_G)\mathbf{\Omega}\right] - \log|\mathbf{\Omega}|.$$

This objective function can be iteratively minimized for both matrix parameters. In any given iteration, we first solve of $\hat{\mathbf{B}}_G$ with a fixed $\mathbf{\Omega}$ using coordinate descent. Then, we can solve for $\hat{\mathbf{\Omega}}$ with the fixed $\hat{\mathbf{B}}_G$ at the given iteration with graphical lasso. We iterate until the convergence tolerance parameter is met. Full details are outlined in Rothman et al[13].

Multivariate elastic net regression using stacked generalization

We employ Rauschenberger and Glaab's `joinet` R package[14] that uses a stacked generalization for multivariate elastic net regression. In general, `joinet` has two steps for prediction:

1. In the first step, or layer, each column of $\mathbf{Y}_G^*$ is predicted from $\mathbf{X}_G$ using elastic net regression via cross-validation to prevent data leakage. This gives us a predicted vector $Y_{g,m}^{(cv)}$ of isoform expression for each isoform m, and taken together, a predicted matrix of isoform expressions $\mathbf{Y}_G^{(cv)}$.
2. In the second layer, each column of $\mathbf{Y}_G^*$ is predicted from $\mathbf{Y}_G^{(cv)}$ with LASSO regression.

Through this two-step prediction, we can estimate a matrix of predicted SNP-isoform effects $\hat{\mathbf{B}}_G$. For each SNP, this stacking process exchanges information among the estimated effects on the isoforms, such that the final estimated effect one a single isoform combines the initial SNP effect estimates on all isoforms linearly.

Sparse partial least squares

Lastly, we use sparse partial least squares[15], as implemented in the `spls` R package. First, it is important to note that partial least squares is a alternative to ordinary least squares for linear regression models without proper conditions. Partial least squares hinges on a dimension reduction technique that assumes that there is a latent decomposition of the response matrix (matrix of isoform expression in the case of isoTWAS) and the predictor matrix (the design matrix of SNPs). This latent decomposition is represented with a K-dimensional matrix $\mathbf{T}$. Partial least squares estimates $\mathbf{T} = \mathbf{X}_G\mathbf{W}$ through successive optimization steps to find the columns of $\mathbf{W}$ using an objective function that depends on the columns of $\mathbf{W}$ and the covariance between the response and predictor matrices.

Sparse partial least squares identifies this latent decomposition with added parameters to induce sparsity. In short, let $\mathbf{M} = \mathbf{X}_G'\mathbf{Y}_G^*\mathbf{Y}_G'^*\mathbf{X}_G$. Sparse partial least squares attempts to minimize the following objective function for ω and c, subject to $\omega'\omega = 1$:

$$-\kappa\omega'\mathbf{M}\omega + (1-\kappa)(c-\omega)'\mathbf{M}(c-\omega) + \lambda_1\|c\|_1 + \lambda_2\|c\|_2^2.$$

There are four parameters that require tuning through cross-validation ($\kappa, \lambda_1, \lambda_2, K$). In isoTWAS, we find the optimal $\kappa \in \{0.1, 0.2, 0.3, \dots, 0.9\}$, $K \in \{1, 2, \dots, \lfloor M/2 \rfloor\}$, λ_1 and λ_2 through 5-fold cross-validation.

The isoTWAS package also allows the user to fit a univariate model for each isoform. We use this as a baseline to compare the advantages of multivariate modelling to this univariate approach.

Univariate modeling

The simplest method implemented is univariate predictive modelling, as implemented in Gusev et al's FUSION software[16]. We ignore the correlation structure between isoforms and train a univariate model. For the mth isoform, we fit:

$$y^*_{G,m} = \mathbf{X}_G \beta_{G,m} + \epsilon_{G,m} \tag{2}$$

We include three univariate methods:

1. **Elastic net regression with elastic net mixing parameter** $\alpha = 0.5$[12]. This procedure finds the $\hat{\beta}_{G,m}$ that minimizes

$$L(\beta_{G,m}) = \frac{1}{2N} \sum_{i=1}^{N} (y_{G,m,i} - x^T_{G,i}\beta_{G,m})^2 + \lambda[(1-\alpha)\|\beta_{G,m}\|_2^2/2 + \alpha\|\beta_{G,m}\|_1].$$

   We use the `glmnet` package in `R` for implementation with cross-validation.

2. **Best linear unbiased predictor (BLUP) using a linear mixed model**[17]. Here, we assume, in Equation 2, that $\beta_{G,m}$ are random SNP effects on the isoform m, such that $\beta_{G,m} \sim \mathbf{N}\left(\mathbf{0}, \frac{\sigma^2_m}{P} I_N\right)$. Here, σ^2_m is a variance parameter for the SNP effects. We can calculate the BLUP of $\beta_{G,m}$ with the following solution of the Henderson mixed-model[17]:

$$\hat{\beta}_{G,m} = \frac{\hat{\sigma}^2_m}{M} \mathbf{X}^T_G \hat{\mathbf{V}}^{-1} y^*_{G,m},$$

   where $\hat{\sigma}^2_m$ and $\mathbf{V} = \sigma^2_m \mathbf{X}_G \mathbf{X}^T_G / P + \sigma^2_\epsilon \mathbf{I}_N$ are estimated with restricted maximum likelihood estimation and subsequent matrix multiplication. We implement an estimation to this model using ridge regression with the `rrBLUP`package in `R`.

3. **Sum of Single Effects (SuSiE) regression.** Here, we assume that, in Equation 2, $\beta_{G,m} = \sum_{i=1}^{L} \beta_{l,G,m}$, where $\beta_{l,G,m}$ has exactly one non-zero element. SuSiE estimates the variance components using maximum likelihood prior to the estimating $\beta_{G,m}$ using an empirical Bayes approach. We implement this procedure using the `susieR` package in `R`[18].

Association testing procedure

We employ a stage-wise testing procedure, similar to the `stageR` method[19].

1. We impute genetically-regulated expression of each isoform and estimate associations between each isoform using (1) the appropriate linear regression if we have access to individual-level genotypes in the GWAS and (2) the weighted burden test if we only have access to GWAS summary statistics[16]. We use an LD reference panel that appropriately matches the ancestry of the GWAS sample and the eQTL sample the predictive models were trained with.

2. Given the Wald-type test statistics $Z_1, \ldots, Z_m$ for a given gene, we run an omnibus test to aggregate the test statistics of isoforms of the same gene. We employ either (1) minimum P-value aggregation (i.e. set the gene-level omnibus P-value to the minimum isoform-level P-value), (2) an aggregated Cauchy association test (ACAT)[20], or (3) Chi-square aggregation, where we define the gene-level test statistic $T_G = \sum_{i=1}^{m} Z_i^2$ and compare to the Chi-square distribution with m degrees of freedom. We correct for multiple comparisons using the Benjamini-Hochberg procedure.

3. We then run an isoform-level multiple testing procedure using the Shaffer MSRB method to assess all isoform-level associations[19]. This procedure controls the family-wide error rate when hypotheses are correlated within the family (i.e. isoforms of the same gene).

Given any overlapping isoforms (i.e. isoforms within 0.5 Megabases of one another), we use transcript-level probabilistic fine-mapping[21] to generate a 90% credible set of associated isoforms.

Simulation framework and parameters

Here, we adopt techniques from Mancuso et al's `twas_sim` package[22] to simulate multivariate isoform expression. We consider the following model

$$\mathbf{Y} = \mathbf{XB} + \mathbf{U} + \epsilon,$$

where, for n total samples, $\mathbf{Y}$ is an $n \times m$ matrix of expression values for m isoforms, $\mathbf{X}$ is an $n \times p$ matrix of p SNPs within 1 Megabase of the isoforms in $\mathbf{Y}$, $\mathbf{B}$ is an $p \times m$ matrix of SNP-isoform effects, $\mathbf{U}$ is the non-cis genetic effects on isoforms that are correlated between both isoforms and samples, and ϵ represents the independent noise added to each isoform separately. We first simulate the SNPs in $\mathbf{X}$ by selecting all the SNPs within 1 Megabase of 22 randomly selected genes (1 per chromosome), by using the linkage disequilibrium matrix from European samples of the 1000 Genomes Project and the framework outlined in `twas_sim`. We then simulate $\mathbf{B}$ by selecting p_c proportion of the SNPs in $\mathbf{X}$ as "causal" and generating a non-zero effect size for these SNPs. We allow for a proportion, p_s, of these "causal" SNPs to be shared across different isoforms. For example, if we set $p_s = 0.50$, we select $0.5p_c$ of the SNPs to be shared across all isoforms and assign, for each isoform, a non-zero effect for these selected shared SNPs. For each isoform, an additional $0.5p_c$ proportion of the SNPs will be randomly selected as non-zero effect SNPs. We then scale each column of $\mathbf{B}$ to ensure that the genetically-determined portion of each column of $\mathbf{Y}$ equals the isoform expression heritability parameter h_g^2.

Next, we simulate $U \sim MVN(\mathbf{0}, \sigma_h V, \sigma_h W)$. σ_h is a tunable parameter for controlling the proportion of variance in isoform expression explained, and V and W are correlation matrices between isoforms and samples, respectively. As simulating positive-semidefinite matrices, especially of large dimension, is difficult, we employ a heuristic that roughly generates dense correlation matrices (off-diagonals are far from 0) for V and sparser correlation matrices (off-diagonals are closer to 0) for W. For V and W, we first generate V_1 , an $m \times m$ matrix, and W_1, an $n \times n$ matrix, where each off-diagonal element is drawn from Unif$(-.5, .5)$ or Unif$(-0.02, 0.02)$, respectively, and the diagonal is set to 1. We then set $V = V_1'V_1/max(V_1)$ and $W = W_1'W_1/max(W_1)$. Lastly, we draw $\epsilon_i \sim N(\mathbf{0}, \sigma_e^2 I)$, where $\sigma_e^2 = 1 - \sigma_h - h_g^2$.

We conduct these simulations 10,000 times across the following set of parameters:

- $n \in \{200, 500, 1000\}$
- $p_c \in \{0.001, 0.01, 0.05\}$
- $h_g^2 \in \{0.05, 0.10, 0.25\}$
- $p_s \in \{0, 0.5, 1\}$
- $\sigma_h \in \{0.1, 0.25\}$

For the GWAS dataset, we first generate genotypes and genetically-regulated isoform expression using the same framework as the QTL dataset and the same causal $\mathbf{B}$ matrix. We then estimate traits in 3 scenarios with a GWAS sample size of 50,000:

1. **Only gene-level expression has a non-zero effect on trait.** Here, we sum the isoform expression to generate a simulated gene expression. We randomly simulate the effect size and scale the error to ensure trait heritability $h_t^2 \in \{0.01, 0.05, 0.10\}$.

2. **Only 1 isoform has a non-zero effect on the trait.** Here, we generate a multivariate isoform expression matrix with 2 isoforms and scale the total gene expression value such that one isoform (called the effect isoform) makes up $p_g \in \{0.10, 0.30, 0.50, 0.70, 0.90\}$ proportion of total gene expression. We then generate effect size for one of the isoforms and scale the error to ensure trait heritability $h_t^2 \in \{0.01, 0.05, 0.10\}$.

3. **Two isoforms with different effects on traits**. Here, we generate a multivariate isoform expression matrix with 2 isoforms that make up equal portions of the total gene expression. We then generate an effect size of α for one isoform and $p_e\alpha$ for the other isoform, such that $p_e \in \{-1, -0.5, -0.2, 0.2, 0.5, 1\}$. We then scale the error to ensure trait heritability $h_t^2 \in \{0.01, 0.05, 0.10\}$.

We also benchmark transcript-level fine-mapping using FOCUS[21]. Here, we use a similar framework, as above. We simulate a gene with 5 or 10 isoforms with the same QTL architecture parameters. We randomly selected one of the isoforms to be the "causal" effect isoform on the trait in Scenario 2 above. Then, we run transcript-level fine-mapping using FOCUS and record the size of the 90% credible set of isoforms and the sensitivity of the 90% credible set (i.e., the proportion of credible sets that contain the "causal" isoform).

Supplementary Table Legends

Table S1: Sample size, source, and tissues for functional genomics reference panels

Table S2: Number of genes/models that pass cross-validation prediction cutoffs using TWAS and isoTWAS feature selection criteria and prediction methods. Data here underlies Figure 3 and Extended Data Figures 3-5.

Table S3: Distribution of predictive external R^2 of observed total gene expression vs. predicted total gene expression (isoTWAS -TWAS). Data here underlies Figure 3.

Table S4: Number of GWAS loci with an isoTWAS-, TWAS-, and splice-TWAS-prioritized gene within 0.5 Mb. Data here underlies Figure 5.

Table S5: TWAS and fine-mapping results for genes with adjusted $P < 0.05$ and permutation $P < 0.05$ across 15 traits using adult brain cortex models. Data here underlies Figure 5.

Table S6: isoTWAS and fine-mapping results for genes with adjusted $P < 0.05$ and permutation $P < 0.05$ across 15 traits using adult brain cortex models. Data here underlies Figure 5.

Table S7: TWAS and fine-mapping results for genes with adjusted $P < 0.05$ and permutation $P < 0.05$ across 15 traits using developmental brain cortex models. Data here underlies Figure 5.

Table S8: isoTWAS and fine-mapping results for genes with adjusted $P < 0.05$ and permutation $P < 0.05$ across 15 traits using developmental brain cortex models. Data here underlies Figure 5.

Table S9: Empirical bayes estimates of test statistic inflation and increase in χ^2 test statistics of gene-level associations using isoTWAS and TWAS. Data here underlies Figure 5.

Table S10: Accession numbers and URLs for data access

Supplementary Data Legends

Data 1: Predictive performance comparison of isoTWAS multivariate methods in simulated data across a variety of genetic architecture settings. Data here underlies Extended Data Figure 2.

Data 2: Predictive performance comparison of isoTWAS and TWAS gene expression prediction in simulated data across a variety of genetic architecture settings. Data here underlies Figure 2 and Extended Data Figure 2.

Data 3: Isoform expression prediction metrics across a variety of factors, using 48 GTEx datasets. Data here underlies Extended Data Figure 6.

Data 4: Gene expression prediction metrics across a variety of factors, using 48 GTEx datasets. Data here underlies Extended Data Figure 6.

Data 5: False positive rates using isoTWAS and TWAS to detect a gene-trait association at P < 0.05 across a variety genetic architecture parameters. Data here underlies Extended Data Figure 7.

Data 6: Power to detect trait association at $P < 2.5 \times 10^{-6}$ across 1,000 simulations each for 22 genes using TWAS and isoTWAS across various simulations. These simulations are under Scenario 1 in Fig. 4a (gene has a true effect on the trait, but none of the isoforms have a true effect on the trait). Data here underlies Figure 4 and Extended Data Figure 7.

Data 7: Power to detect trait association at $P < 2.5 \times 10^{-6}$ across 1,000 simulations each for 22 genes using TWAS and isoTWAS (ACAT) across various simulations. These simulations are under Scenario 2 in Fig. 4b (a gene has multiple isoforms, only one has an effect on the trait, and we vary the usage of this effect isoform). Data here underlies Figure 4 and Extended Data Figure 7.

Data 8: Power to detect trait association at P < 2.5 x 10^{-6} across 1,000 simulations each for 22 genes using TWAS and isoTWAS (ACAT) across various simulations. These simulations are under Scenario 3 in Fig. 4c (a gene has two isoforms with differing effects on the trait, and we vary the effect size of one of the isoforms). Data here underlies Figure 4 and Extended Data Figure 7.

Data 9: Sensitivity and mean set size of 90% credible sets determined by FOCUS in simulated data across a variety of genetic architecture parameters. Data here underlies Extended Data Figure 7.

Data 10: Raw TWAS results across 15 neuropsychiatric traits using adult brain cortex expression models. Data here underlies Extended Data Figure 8-9.

Data 11: Raw isoTWAS results across 15 neuropsychiatric traits using adult brain cortex expression models. Data here underlies Extended Data Figure 8-9.

Data 12: Raw TWAS results across 15 neuropsychiatric traits using developmental brain cortex expression models. Data here underlies Extended Data Figure 8-9.

Data 13: Raw isoTWAS results across 15 neuropsychiatric traits using developmental brain cortex expression models. Data here underlies Extended Data Figure 8-9.

Data 14: GWAS and nominal eQTL and isoQTL summary statistics corresponding to isoTWAS isoform-trait association examples shown in Fig. 6 and Extended Data Fig. 10b. Data here underlies Figure 6 and Extended Data Figure 10.

Supplementary References

1. Vitting-Seerup, K. & Sandelin, A. IsoformSwitchAnalyzeR: Analysis of changes in genome-wide patterns of alternative splicing and its functional consequences. *Bioinformatics* **35**, 4469–4471 (2019).
2. Soneson, C., Love, M. I. & Robinson, M. D. Differential analyses for RNA-seq: Transcript-level estimates improve gene-level inferences. *F1000Research 2015 4:1521* **4**, 1521–1521 (2016).
3. Patro, R., Duggal, G., Love, M. I., Irizarry, R. A. & Kingsford, C. Salmon provides fast and bias-aware quantification of transcript expression. *Nature Methods* **14**, 417–419 (2017).
4. Love, M. I. *et al.* Tximeta: Reference sequence checksums for provenance identification in RNA-seq. *PLOS Computational Biology* **16**, e1007664–e1007664 (2020).
5. Schrode, N. *et al.* Synergistic effects of common schizophrenia risk variants. *Nature genetics* **51**, 1475–1485 (2019).
6. Bhattacharjee, S. *et al.* A Subset-Based Approach Improves Power and Interpretation for the Combined Analysis of Genetic Association Studies of Heterogeneous Traits. *American Journal of Human Genetics* **90**, 821–835 (2012).
7. O'Donnell-Luria, A. H. *et al.* Heterozygous Variants in KMT2E Cause a Spectrum of Neurodevelopmental Disorders and Epilepsy. *American Journal of Human Genetics* **104**, 1210–1222 (2019).
8. Reay, W. R. & Cairns, M. J. Pairwise common variant meta-analyses of schizophrenia with other psychiatric disorders reveals shared and distinct gene and gene-set associations. *Translational Psychiatry* **10**, 1–11 (2020).
9. Nishioka, K. *et al.* PR-Set7 Is a Nucleosome-Specific Methyltransferase that Modifies Lysine 20 of Histone H4 and Is Associated with Silent Chromatin. *Molecular Cell* **9**, 1201–1213 (2002).
10. Trubetskoy, V. *et al.* Mapping genomic loci implicates genes and synaptic biology in schizophrenia. *Nature* **604**, 502–508 (2022).
11. Schmidt-Kastner, R., Guloksuz, S., Kietzmann, T., Os, J. van & Rutten, B. P. F. Analysis of GWAS-derived schizophrenia genes for links to ischemia-hypoxia response of the brain. *Frontiers in Psychiatry* **11**, 393 (2020).
12. Friedman, J., Hastie, T. & Tibshirani, R. Regularization Paths for Generalized Linear Models via Coordinate Descent. *Journal of Statistical Software* **33**, 1–22 (2010).
13. Rothman, A. J., Levina, E. & Zhu, J. Sparse multivariate regression with covariance estimation. *Journal of Computational and Graphical Statistics* **19**, 947–962 (2010).
14. Rauschenberger, A. & Glaab, E. Predicting correlated outcomes from molecular data. *Bioinformatics* **37**, 3889–3895 (2021).
15. Chun, H. & Keleş, S. Sparse partial least squares regression for simultaneous dimension reduction and variable selection. *Journal of the Royal Statistical Society. Series B, Statistical Methodology* **72**, 3–25 (2010).
16. Gusev, A. *et al.* Integrative approaches for large-scale transcriptome-wide association studies. *Nature Genetics* **48**, 245–252 (2016).
17. Endelman, J. B. Ridge Regression and Other Kernels for Genomic Selection with R Package rrBLUP. *The Plant Genome* **4**, 250–255 (2011).
18. Wang, G., Sarkar, A., Carbonetto, P. & Stephens, M. A simple new approach to variable selection in regression, with application to genetic fine mapping. *Journal of the Royal Statistical Society: Series B* **82**, 1273–1300 (2020).
19. Van den Berge, K., Soneson, C., Robinson, M. D. & Clement, L. stageR: A general stage-wise method for controlling the gene-level false discovery rate in differential expression and differential transcript usage. *Genome Biology 2017 18:1* **18**, 1–14 (2017).
20. Liu, Y. *et al.* ACAT: A Fast and Powerful p Value Combination Method for Rare-Variant Analysis in Sequencing Studies. *American Journal of Human Genetics* **104**, 410–421 (2019).

21. Mancuso, N. *et al.* Probabilistic fine-mapping of transcriptome-wide association studies. *Nature Genetics* **51**, 675–682 (2019).
22. Wang, X., Lu, Z., Bhattacharya, A., Pasaniuc, B. & Mancuso, N. Twas_sim, a Python-based tool for simulation and power analysis of transcriptome-wide association analysis. *Bioinformatics* **39**, btad288 (2023).
