# Supplementary material for: Isoform-level transcriptome-wide association uncovers genetic risk mechanisms for neuropsychiatric disorders in the human brain
Source: Nat Genet. 2023 Nov 30;55(12):2117–28. doi: 10.1038/s41588-023-01560-2 (PMC10703692; doi:10.1038/s41588-023-01560-2)
Supplement: Supplementary file 2 — Reporting Summary [file 41588_2023_1560_MOESM2_ESM.pdf]

nature portfolio

Corresponding author(s): Arjun Bhattacharya

Last updated by author(s): Sep 8, 2023

Reporting Summary

Nature Portfolio wishes to improve the reproducibility of the work that we publish. This form provides structure for consistency and transparency in reporting. For further information on Nature Portfolio policies, see our Editorial Policies and the Editorial Policy Checklist.

Statistics

For all statistical analyses, confirm that the following items are present in the figure legend, table legend, main text, or Methods section.

n/a	Confirmed	
☐	☒	The exact sample size (*n*) for each experimental group/condition, given as a discrete number and unit of measurement
☐	☒	A statement on whether measurements were taken from distinct samples or whether the same sample was measured repeatedly
☐	☒	The statistical test(s) used AND whether they are one- or two-sided<br>*Only common tests should be described solely by name; describe more complex techniques in the Methods section.*
☐	☒	A description of all covariates tested
☐	☒	A description of any assumptions or corrections, such as tests of normality and adjustment for multiple comparisons
☐	☒	A full description of the statistical parameters including central tendency (e.g. means) or other basic estimates (e.g. regression coefficient) AND variation (e.g. standard deviation) or associated estimates of uncertainty (e.g. confidence intervals)
☐	☒	For null hypothesis testing, the test statistic (e.g. *F*, *t*, *r*) with confidence intervals, effect sizes, degrees of freedom and *P* value noted<br>*Give P values as exact values whenever suitable.*
☐	☒	For Bayesian analysis, information on the choice of priors and Markov chain Monte Carlo settings
☐	☒	For hierarchical and complex designs, identification of the appropriate level for tests and full reporting of outcomes
☐	☒	Estimates of effect sizes (e.g. Cohen's *d*, Pearson's *r*), indicating how they were calculated

Our web collection on statistics for biologists contains articles on many of the points above.

Software and code

Policy information about availability of computer code

Data collection	No software was used for data collected.
Data analysis	The following software was used: Salmon v1.5.2, Salmon v1.8.0, tximeta v1.16.1, DESeq2 v1.38.3, fishpond v2.4.1, IsoformSwitchAnalyzeR v1.20.0, edgeR v3.40.2, minimac4, eagle v2.4, CrossMap v.0.6.3, PLINK v1.90b6.21, bcftools v1.11, samtools v1.14, PicardTools v2.25.0, QTLtools v1.3.1. isoTWAS is available as an R package at https://github.com/bhattacharya-a-bt/isotwas (isoTWAS v1.0.0). Sample scripts for analyses are available at https://github.com/bhattacharya-a-bt/isotwas_manu_scripts.

For manuscripts utilizing custom algorithms or software that are central to the research but not yet described in published literature, software must be made available to editors and reviewers. We strongly encourage code deposition in a community repository (e.g. GitHub). See the Nature Portfolio guidelines for submitting code & software for further information.

Data

Policy information about availability of data

All manuscripts must include a data availability statement. This statement should provide the following information, where applicable:

- Accession codes, unique identifiers, or web links for publicly available datasets
- A description of any restrictions on data availability
- For clinical datasets or third party data, please ensure that the statement adheres to our policy

GTEx genetic, transcriptomic, and covariate data were obtained through dbGAP approval at accession number phs000424.v8.p2. Linkage disequilibrium reference

data from the 1000 Genomes Project were obtained at this link: https://www.internationalgenome.org/data-portal/sample. GENCODE reference transcriptome and assembly was downloaded from https://www.gencodegenes.org/human/release_38.html with GenBank assembly accession GCA_000001405.28. GWAS summary statistics were obtained at the following links: ADHD (https://www.med.unc.edu/pgc/download-results/), ALZ (https://ctg.cncr.nl/software/summary_statistics/), AN (http://www.med.unc.edu/pgc/results-and-downloads), ASD (https://www.med.unc.edu/pgc/download-results/), BP (https://www.med.unc.edu/pgc/download-results/), BV (https://ctg.cncr.nl/software/summary_statistics), CDG (https://www.med.unc.edu/pgc/results-and-downloads), CortTH (https://enigma.ini.usc.edu/research/download-enigma-gwas-results/), ICV (https://enigma.ini.usc.edu/research/download-enigma-gwas-results/), MDD (http://dx.doi.org/10.7488/ds/2458), NTSM (https://ctg.cncr.nl/software/summary_statistics/neuroticism_summary_statistics), OCD (https://www.med.unc.edu/pgc/download-results/), PANIC (https://www.med.unc.edu/pgc/download-results/), PTSD (https://www.med.unc.edu/pgc/results-and-downloads/), and SCZ (https://www.med.unc.edu/pgc/download-results/). The Developmental Brain RNA-seq and genotype dataset from Walker et al is available at dbGAP with accession number phs001900. The subset of Adult Brain RNA-seq and genotype data from the PsychENCODE Consortium is available at https://psychencode.synapse.org/DataAccess and from AMP-AD is available at https://adknowledgeportal.synapse.org/Data%20Access. GWAS summary statistics and accession numbers to genotype and RNA-seq data are provided in Supplementary Table S10. isoTWAS models for 48 tissues from GTEx are available at https://zenodo.org/record/8047940, adult brain cortex from PsychENCODE and AMP-AD are available at https://zenodo.org/record/8048198, and developmental brain cortex from Walker et al are available at https://zenodo.org/record/8048137.

Research involving human participants, their data, or biological material

Policy information about studies with human participants or human data. See also policy information about sex, gender (identity/presentation), and sexual orientation and race, ethnicity and racism.

Reporting on sex and gender	Only publicly available data was used, as linked in the Data Availability statement. No new data was collected in this study.
Reporting on race, ethnicity, or other socially relevant groupings	N/A
Population characteristics	N/A
Recruitment	N/A
Ethics oversight	N/A

Note that full information on the approval of the study protocol must also be provided in the manuscript.

Field-specific reporting

Please select the one below that is the best fit for your research. If you are not sure, read the appropriate sections before making your selection.

☒ Life sciences ☐ Behavioural & social sciences ☐ Ecological, evolutionary & environmental sciences

For a reference copy of the document with all sections, see nature.com/documents/nr-reporting-summary-flat.pdf

Life sciences study design

All studies must disclose on these points even when the disclosure is negative.

Sample size	This study includes an integrative data analysis of data from the Genotype Tissue-Expression Project. Samples sizes for this dataset are reported in Aguet et al 2020, Science and in Supplementary Table S1. We also include data from the PsychENCODE Consortium and AMP-AD Consortium using prefrontal cortex RNA-seq and genotype data for two samples: adult (N = 2,115) and developmental frontal cortex (N = 205). Maximal sample sizes depended on the number of samples with both RNA-seq and genotype data; no power calculations were conducted to pre-determine sample size. We conducted gene- and isoform-level trait mapping for 15 neuropsychiatric traits: attention-deficit hyperactivity disorder (ADHD, Ncases = 20,183/Ncontrols = 35,191), Alzheimer's disease (ALZ, 90,338/1,036,225), anorexia nervosa (AN, 16,992/55,525), autism spectrum disorder (ASD, 18,381/27,969), bipolar disorder (BP, 41,917/371,549), brain volume (BV, N=47,316), cross-disorder (CDG, 232,964/494,162), cortical thickness (CortTH, N = 51,665), intracranial volume (ICV, N = 32,438), major depressive disorder (MDD, 246,363/561,190), neuroticism (NTSM, N = 449,484), obsessive compulsive disorder (OCD, 2,688/7,037), panic and anxiety disorders (PANIC, 2,248/7,992), post-traumatic stress disorder (PTSD, 32,428/174,227), and schizophrenia (SCZ, 69,369/236,642). These 15 traits represent complex brain-related traits that are studied by large consortium with sufficient sample sizes (Psychiatric Genomics Consortium, Complex Traits Genomics Lab, etc). These 15 traits represent a wide range of brain-related disorders and traits that provide a comprehensive analysis of the utility of isoform-level TWAS trait mapping.
Data exclusions	GTEx data was restricted to individuals of European genetic ancestry to ensure portability of genetic predictions. PsychENCODE and AMP-AD individuals were removed if their WGCNA network connectivity scores based on isoform-level expression was less than -3; these low scores indicate that these samples may be plagued by technical biases that may affect the estimation of genetic effects on gene- and isoform-level expression. These data exclusion factors were pre-determined.
Replication	There are no experimental findings to replicate.
Randomization	There are no experimental groups in this study.
Blinding	No data collection was conducted in this study. The data used in this study has been previously reported. As such, the investigators of this work were blinded to group allocation.

Reporting for specific materials, systems and methods

We require information from authors about some types of materials, experimental systems and methods used in many studies. Here, indicate whether each material, system or method listed is relevant to your study. If you are not sure if a list item applies to your research, read the appropriate section before selecting a response.

Materials & experimental systems

n/a	Involved in the study
☒	☐ Antibodies
☒	☐ Eukaryotic cell lines
☒	☐ Palaeontology and archaeology
☒	☐ Animals and other organisms
☒	☐ Clinical data
☒	☐ Dual use research of concern
☒	☐ Plants

Methods

n/a	Involved in the study
☒	☐ ChIP-seq
☒	☐ Flow cytometry
☒	☐ MRI-based neuroimaging
